# Regional distribution of acoustic-based lung vibration as a function of mechanical ventilation mode

**DOI:** 10.1186/cc5706

**Published:** 2007-02-22

**Authors:** R Phillip Dellinger, Smith Jean, Ismail Cinel, Christina Tay, Susmita Rajanala, Yael A Glickman, Joseph E Parrillo

**Affiliations:** 1 Division of Cardiovascular Disease and Critical Care Medicine, Robert Wood Johnson School of Medicine, University of Medicine and Dentistry of New Jersey, Cooper University Hospital, 1 Cooper Plaza, Dorrance Building, Suite 393, Camden, NJ 08103, USA; 2 Deep Breeze Ltd. 2 Hailan St., P.O. Box 140, North Industrial Park, Or-Akiva, 30600, Israel

## Abstract

**Introduction:**

There are several ventilator modes that are used for maintenance mechanical ventilation but no conclusive evidence that one mode of ventilation is better than another. Vibration response imaging is a novel bedside imaging technique that displays vibration energy of lung sounds generated during the respiratory cycle as a real-time structural and functional image of the respiration process. In this study, we objectively evaluated the differences in regional lung vibration during different modes of mechanical ventilation by means of this new technology.

**Methods:**

Vibration response imaging was performed on 38 patients on assist volume control, assist pressure control, and pressure support modes of mechanical ventilation with constant tidal volumes. Images and vibration intensities of three lung regions at maximal inspiration were analyzed.

**Results:**

There was a significant increase in overall geographical area (*p *< 0.001) and vibration intensity (*p *< 0.02) in pressure control and pressure support (greatest in pressure support), compared to volume control, when each patient served as his or her own control while targeting the same tidal volume in each mode. This increase in geographical area and vibration intensity occurred primarily in the lower lung regions. The relative percentage increases were 28.5% from volume control to pressure support and 18.8% from volume control to pressure control (*p *< 0.05). Concomitantly, the areas of the image in the middle lung regions decreased by 3.6% from volume control to pressure support and by 3.7% from volume control to pressure control (*p *< 0.05). In addition, analysis of regional vibration intensity showed a 35.5% relative percentage increase in the lower region with pressure support versus volume control (*p *< 0.05).

**Conclusion:**

Pressure support and (to a lesser extent) pressure control modes cause a shift of vibration toward lower lung regions compared to volume control when tidal volumes are held constant. Better patient synchronization with the ventilator, greater downward movement of the diaphragm, and decelerating flow waveform are potential physiologic explanations for the redistribution of vibration energy to lower lung regions in pressure-targeted modes of mechanical ventilation.

## Introduction

There are several ventilator modes that are more commonly used for maintenance mechanical ventilation (MV) of the intensive care unit (ICU) patient [1,2]. These include assist volume control (VC), assist pressure control (PC), and pressure support (PS) modes. There is no conclusive evidence that one mode of ventilation is better than another.

With most ventilators, selection of VC requires setting of tidal volume (V_T_), respiratory rate (RR), and inspiratory flow rate or time. In PC mode, pressure, RR, and inspiratory time are set. In PS mode, the level of inspired pressure is set and all other parameters are determined by the patient.

The major differences between VC and the other two modes are the inspiratory flow and pressure waveforms [3-5]. In VC mode, the pressure rises throughout inspiration and the inspiratory flow can be constant, decelerating, or sine-patterned. On the other hand, both PC and PS have a square pressure waveform and a decelerating inspiratory flow pattern, in which the inspiratory flow rate is high at the beginning and decreases with time. Although some studies have shown differences in work of breathing [6], lung mechanics [7,8], and gas exchange [8,9] in patients ventilated with these different waveforms, no consistent reproducible findings have demonstrated the benefit of one mode of ventilation over another. In fact, modes are routinely chosen by the personal preference of the treating physician or respiratory therapist.

Vibration response imaging (VRI) is a novel technology that measures vibration energy of lung sounds generated during the respiratory process. As air moves in and out of the lungs, the vibrations propagate through the lung tissue and are recorded by 36 surface skin sensors, which are spatially distributed and attached to the patient's back. The vibration energy is transmitted to the VRI device, and a dynamic digital image is created by means of specifically designed proprietary software. An image is displayed using a gray-scale level (similar to ventilation scanning images of the lung), but in contrast to radiolabeled ventilation scanning, VRI technology is non-invasive and does not require the addition of a tracer to either the inspired air or bloodstream. The transmission of an acoustic signal through the lungs is affected by air content and tissue properties [10], and the ability to image the lungs by means of an acoustic signal has been previously demonstrated [11,12].

In the present study, we compare the vibration generated by airflow in a lung ventilated with three different modes of MV: VC, PC, and PS. Validation of the capability of VRI technology to track changes in lung airflow and of the effect of different V_T _values on lung vibration is demonstrated in several subjects. Some of the results included here have been previously reported in our abstracts [13,14].

## Materials and methods

### Patients

The study protocol was approved by the Institutional Review Board, and informed consent was obtained from all patients or their next of kin. Thirty-eight patients (14 men, 24 women) requiring mechanical ventilatory support in the ICU were selected for the study (Table 1). Patients had a mean ± standard deviation (SD) age of 60 ± 16 years, fraction of inspired oxygen (FiO_2_) of 0.41 ± 0.05, and positive end-expiratory pressure (PEEP) of 5.2 ± 0.93 cm H_2_O and were mechanically ventilated for 5 ± 5 days prior to the recordings. Patients were ventilated with one of several types of ventilators: Puritan Bennett 840 (Tyco Healthcare, Mansfield, MA, USA), Servo 900 C, 300, and 300A and the Servo I (Maquet, Inc., Bridgewater, NJ, USA), and Bird 8400 ST (Bird Products Corp., Palm Springs, CA, USA). The selection of initial ventilator mode was decided by the treating physicians and support staff. The relationship between V_T _and flow on lung vibration was demonstrated in four healthy volunteers.

**Table 1:** Patient characteristics (*n *= 38)

	Number (percentage)
Gender	
Male	14 (37%)
Female	24 (63%)
Chest x-ray findings	
Atelectasis	20 (53%)
Pleural effusion	13 (34%)
Cardiomegaly	10 (26%)
Pulmonary edema	2 (5%)
Normal	2 (5%)
Other	6 (16%)
Reason for intubation	
Respiratory distress or failure	27 (71%)
Dyspnea	7 (18%)
Airway protection	3 (8%)
Hypoxia or anoxia	3 (8%)

Patients may have more than one diagnosis and/or radiographic finding.

### Inclusion and exclusion criteria

Patients enrolled in the study were adults (18 to 84 years old) who required minimal to moderate mechanical ventilatory support (peak airway pressure of less than or equal to 30 cm H_2_O, PEEP of less than or equal to 8 cm H_2_O, FiO_2 _of less than or equal to 0.5, and RR of less than or equal to 30 breaths per minute), who had no hypotension or severe hypertension, and whose heart rate was in the acceptable range (that is, 60 to 115 beats per minute). Patients with hemodynamic instability requiring vasopressors, chest cage or spine deformity, or skin lesions or hirsutism on the back and any patient deemed unable to be lifted to a near-sitting position with assistance were excluded. Patients judged to have conditions that would make maintenance of near-constant V_T _difficult (agitation, anxiety, or unstable pulmonary status) were also excluded.

### Study design

No patients were enrolled who were paralyzed or who were sedated to the point of inability to interact with the ventilator. All patients were capable of assisting the ventilator. Three patients were judged as poor candidates for stand-alone PS and were studied in VC and PC modes only. The modes used were as follows:

VC: volume-targeted, time- or patient-triggered (based on the frequency of patient respiratory effort), volume-cycled ventilation with constant flow (square/rectangular inspiratory flow waveform per protocol).

PC: pressure-targeted, time- or patient-triggered (based on the frequency of patient respiratory effort), time-cycled ventilation with variable flow (decelerating) and V_T _maintained near the desired value by pressure adjustment.

PS: all breaths are pressure-targeted and patient-triggered. Flow (decelerating), volume, and inspiratory time could vary based on patient effort, and protocol targets the pressure adjustment to hold V_T _near the desired value.

Because the great majority of ventilated patients included in this study were on VC at the start of the experiment, the first recordings were typically carried out on this mode, followed by PC and then PS. Three patients were unable to trigger the ventilator on PS, so no recording was carried out on this mode. Subgroups of patients who received PC or PS during the first recording (*n *= 3) or who were re-recorded in VC at the end of the study (*n *= 6) were used to assess any effect due to the lack of randomization. When switching from VC to PC and PS, the ventilator was set (pressure adjusted) to achieve the target V_T _delivered in VC mode. Inspiratory time was unchanged from VC to PC and was determined by the patient on PS. V_T_, FiO_2_, and PEEP were held constant.

In addition to the ventilated patients, 20 recordings were performed on four non-intubated healthy volunteers at increasing V_T _values (range 350 to 1,500 ml). This produced steadily increasing flow rates. V_T _values were accurately measured using a CPAP (continuous positive airway pressure) mask and mechanical ventilator. RRs during recordings were kept constant. The sum of the vibration energy in the lungs during each breath cycle (inspiration and expiration) was calculated and matched with each V_T_.

### Recording procedure

The recordings were performed using a VRI device (Deep Breeze Ltd., Or-Akiva, Israel) with two arrays of sensors (six rows by three columns each) or microphones similar to those used in digital stethoscopes. Each array was placed over a lung on the patient's back. The rationale for posterior imaging includes proximity to the lung and difficulty in imaging females anteriorly. To gain access to the patient's back, the patient was lifted to a near-sitting position. The recording was performed during a 20-second period, capturing up to 10 respiratory cycles. Following each recording, the suction was released but the arrays were held in place to ensure no change in array placement for subsequent recordings with different modes. A normalized dynamic image was displayed after each recording, and the raw data were stored digitally on the device for later review and analysis.

The VRI dynamic image is created from a series of gray-scale still images or frames, each of which represents 0.17 seconds of vibration energy recording. The result is a movie depicting a sense of air movement in the lungs. In addition, a graph is produced that represents the average vibration energy as a function of time throughout the respiratory cycle. Artifacts are any distortions in the image which are not related to the condition of the lungs and which are caused by extraneous noises (that is, cough, sneeze, or grunt), vibrations (that is, from stridor or the bed), or excessive motion by the patient during the recording. Artifacts are easily identified in the image, and poor-quality recordings were excluded. Overall, four patients (less than 10%) were excluded due to artifacts. Typical background ICU noise has no effect on VRI recording.

### VRI data analysis

**Figure 1:**
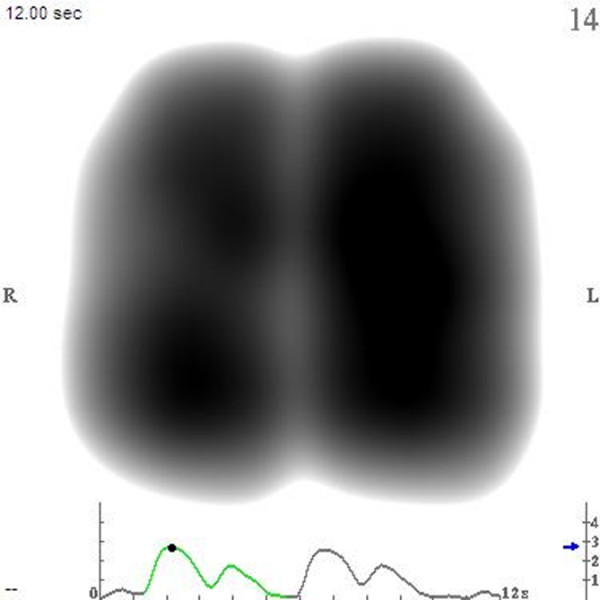
An example of a normal vibration response image. A maximal energy frame from a vibration response image recording of a healthy, 30-year-old, male non-smoker is shown.

Normalization was applied to a predetermined range of frames. Within a frame, the areas with the highest vibration energy are represented as black in a gray-level scale and the areas with the lowest vibration energy are represented as light gray. Areas of a frame are white if their energy is below a signal-to-noise threshold determined by the VRI software. The software displays a video containing those normalized frames in shades of gray which reflect the intensity of vibration at each stage of the respiratory cycle. The maximal energy frame (MEF) is the frame producing the maximal geographical area of lung vibrations in the selected range of frames. In the present study, this frame was used for analysis. Figure 1 is an image from a recording of a 30-year-old, healthy, male non-smoker (video of this recording is available online as Additional file 1). Recordings are saved as both still MEF and dynamic images, which can be analyzed either as a whole or according to specific regions (left, right, upper, middle, and lower lung).

Although a very large amount of information is available within the 20-second recording, it was necessary to select a method of analysis from among various possibilities. Comparisons of MEF areas and vibration energy were preferred techniques because they provide straightforward quantification. MEFs were extracted from normal, regular, and consistent cycles available within each 20-second recording. Artifact-free MEFs were extracted *a priori *from these selected cycles according to predefined rules and criteria listed below. The MEF area of the VRI image was measured using the software ImageJ (National Institute of Health, Bethesda, MD, USA) [15]. Regional areas were obtained by first separating the image into three regions on the basis of the rows of sensors (upper: rows 1 and 2; middle: rows 3 and 4; and lower: rows 5 and 6). Each segment was then measured with ImageJ. Because the position of the sensors was kept the same for each image recorded on a given patient, the three regions were standardized across studies.

The regional vibration energy, which is not affected by normalization of the image, was also analyzed. Vibration intensity is computed in units of energy (watts × constant), reflecting the acoustic energy associated with respiration. The vibration energy was derived from the signal at each of the 36 sensors as follows: the digitized acoustic signals were bandpass-filtered between 150 and 250 Hz to remove heart and muscle sounds; median filtering was applied to suppress impulse noise, and truncation of samples above an automatically determined signal-to-noise threshold was performed. The resulting signal was down-sampled to produce the vibration energy. The regional distribution of vibration energy was also calculated for the frames of interest (MEFs) by means of proprietary software. The percentage changes in vibration energy within the lower lung region (two lower rows of sensors), the middle lung region (two middle rows), and the upper lung region (two upper rows) were calculated and then compared among different modes of MV. The relative percentage changes within the regions of the lung were also assessed to more clearly demonstrate the shift in vibration energy and were also presented.

### Selection of frames for analysis

**Figure 2:**
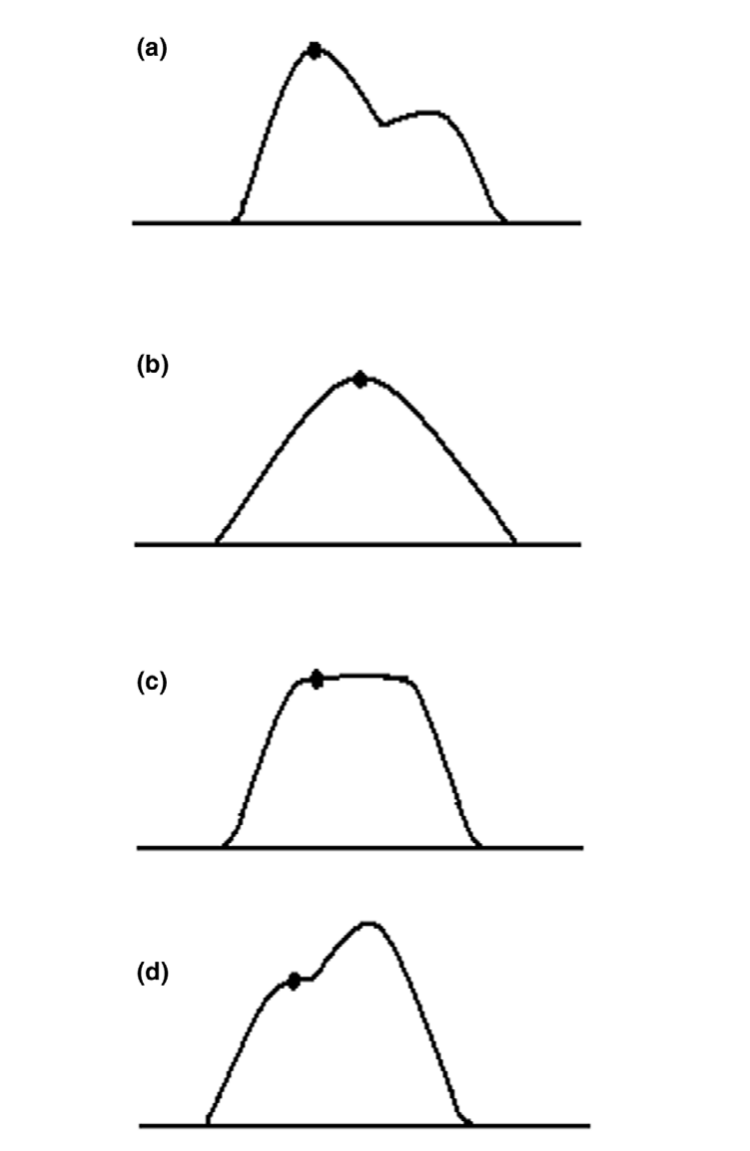
Selection of maximal inspiratory frames for analysis. Examples of frame selection in various vibration response imaging (VRI) waveform patterns are shown. The dot on the VRI waveform represents the area from which the maximal energy frame was chosen for analysis. **(a) **When inspiratory and expiratory vibrations are clearly separated, the maximal energy frame during inspiration (first peak) is chosen. **(b) **When nspiratory and expiratory vibrations merge into one peak, the highest energy frame is chosen. **(c) **When inspiratory and expiratory vibrations form a plateau, first frame at zero slope is chosen. **(d) **When no clear separation exists between inspiratory and expiratory vibrations, and the frame nearest the inflection point of the shoulder is chosen.

Frames were selected *a priori *from the recordings on the basis of the predefined rules and criteria listed below:

1. To correctly characterize respiratory cycles, the following criteria were applied:

- Vibration intensity is lower between two cycles (from expiration to inspiration) than within a same cycle (from inspiration to expiration).

- The distance between expiration and the next inspiration in the VRI energy graph is greater than the distance between inspiration and expiration within the same cycle.

- The area of rapidly increasing vibration from baseline indicates inspiration.

2. To correctly identify inspiration within a respiratory cycle, these criteria were applied:

- The first dramatic rise of vibration in a cycle is inspiration.

- If there is no separation between inspiration and expiration in the VRI energy graph, inspiration is considered to end at the peak signal.

- If there is more than one peak in the cycle, the first peak is considered the maximal inspiration signal.

- If there is a hint of separation in the form of a shoulder in the VRI energy graph, the shoulder is considered an inspiration.

3. These criteria were applied in choosing the maximal inspiration frame (Figure 2):

- The frame with the maximal energy within inspiration is chosen for analysis.

- If inspiration and expiration are clearly separated, the MEF during inspiration (first peak) is chosen (Figure 2a).

- If inspiration and expiration merge into one peak in the waveform, the frame closest to that peak is chosen from the image (Figure 2b).

- If inspiration and expiration form a plateau, the first frame at zero slope is chosen (Figure 2c).

- If there is no peak and the shoulder is curvilinear, the frame nearest the inflection point is chosen (Figure 2d).

4. The following criteria were applied in choosing the range for normalization of recording:

The dynamic image is produced by proprietary software and is normalized based on a chosen range of frames. The MEF at inspiration is selected for analysis.

- The chosen frame must have the highest energy in the range chosen.

- If there is a peak in the waveform, the chosen range consists of the two frames before and two frames after the peak. If this captures a frame with energy greater than the chosen frame, only frames with energy less than the chosen frame are included.

- If there is no peak and only a shoulder, the chosen range consists of the two frames before and the chosen frame.

The program SPSS (SPSS Inc., Chicago, IL, USA) was used for statistical analysis. Mean ± SD or mean ± standard error of the mean (SEM) are reported. Coefficients of determination for linear regression (*R*^2^) were obtained using Microsoft^® ^Office EXCEL 2003 (Microsoft Corporation, Redmond, WA, USA). The Kolmogorov-Smirnov goodness-of-fit test was used to assess the normal distribution of the samples. The Wilcoxon signed ranks test was used to analyze non-normally distributed data, and paired *t *tests were performed for normally distributed data. A *p *value of less than 0.05 was considered statistically significant.

## Results

**Figure 3:**
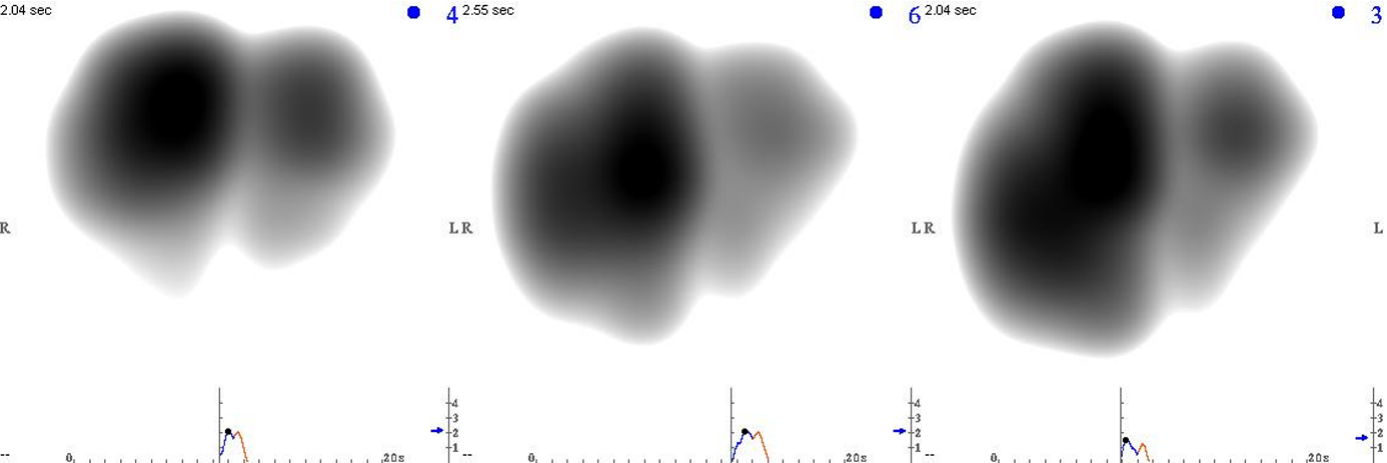
Vibration response images on various modes of mechanical ventilation. Maximal energy frames extracted from recordings of a 73-year-old mechanically ventilated female with respiratory failure secondary to pancreatitis are shown. Chest radiography reported pleural fluid in both lungs. Assist volume control, assist pressure control, and pressure support are shown from left to right. L, left lung; R, right lung.

**Figure 4:**
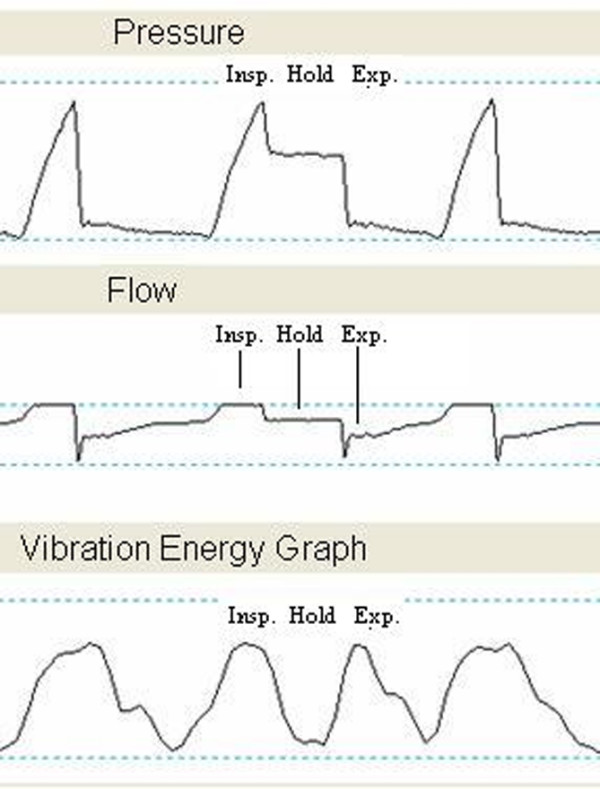
Separation of inspiratory and expiratory signals in a vibration response imaging (VRI) waveform. Separation of inspiratory and expiratory signals produced by application of an inspiratory hold during the second breath in a mechanically ventilated patient is shown. Flow was sampled directly from the ventilator and synchronized with VRI. The three waveforms depict pressure, flow, and vibration as a function of time. Exp., expiratory; Insp., inspiratory.

Successive VRI recordings were performed two to five minutes apart and analyzed from 38 consecutive patients during different modes of MV. Examples of still images of a mechanically ventilated patient on VC, PC, and PS are displayed in Figure 3, and videos of these recordings are available online as Additional files 2, 3, and 4, respectively. There were no differences in RR, heart rate, number of breaths per minute above the set rate, blood pressure, oxygen saturation, PEEP, FiO_2 _values, and V_T _between the three modes (Table 2). Moreover, the phase lag between airflow at the mouth and vibration was minimal (less than 0.2 seconds) as demonstrated by various inspiratory hold experiments (Figure 4).

**Table 2:** Parameters among different modes (*n *= 38)

	VC	PC	PS	*p*
	Mean ± SD	Mean ± SD	Mean ± SD	
		
Tidal volume (ml)	492 ± 86	479 ± 100	439 ± 137	NS
PIP (mm H_2_O)	28 ± 10	25 ± 7	22 ± 8	< 0.05^a^
Respiratory rate (breaths per minute)	21 ± 6	22 ± 6	23 ± 9	NS
Breaths per minute above set respiratory rate	8.0 ± 6.3	8.3 ± 6.3	N/A	NS
Oxygen saturation (percentage)	96 ± 4	96 ± 4	96 ± 3	NS
Heart rate (beats per minute)	90 ± 14	94 ± 14	95 ± 13	NS
Blood pressure (mm Hg)	132/74 ± 23/17	132/75 ± 28/18	131/74 ± 28/19	NS

^a^PIP differed among all three modes. N/A, non-applicable; NS, not significant; PC, assist pressure control; PIP, peak inspiratory pressure; PS, pressure support; SD, standard deviation; VC, assist volume control.

**Figure 5:**
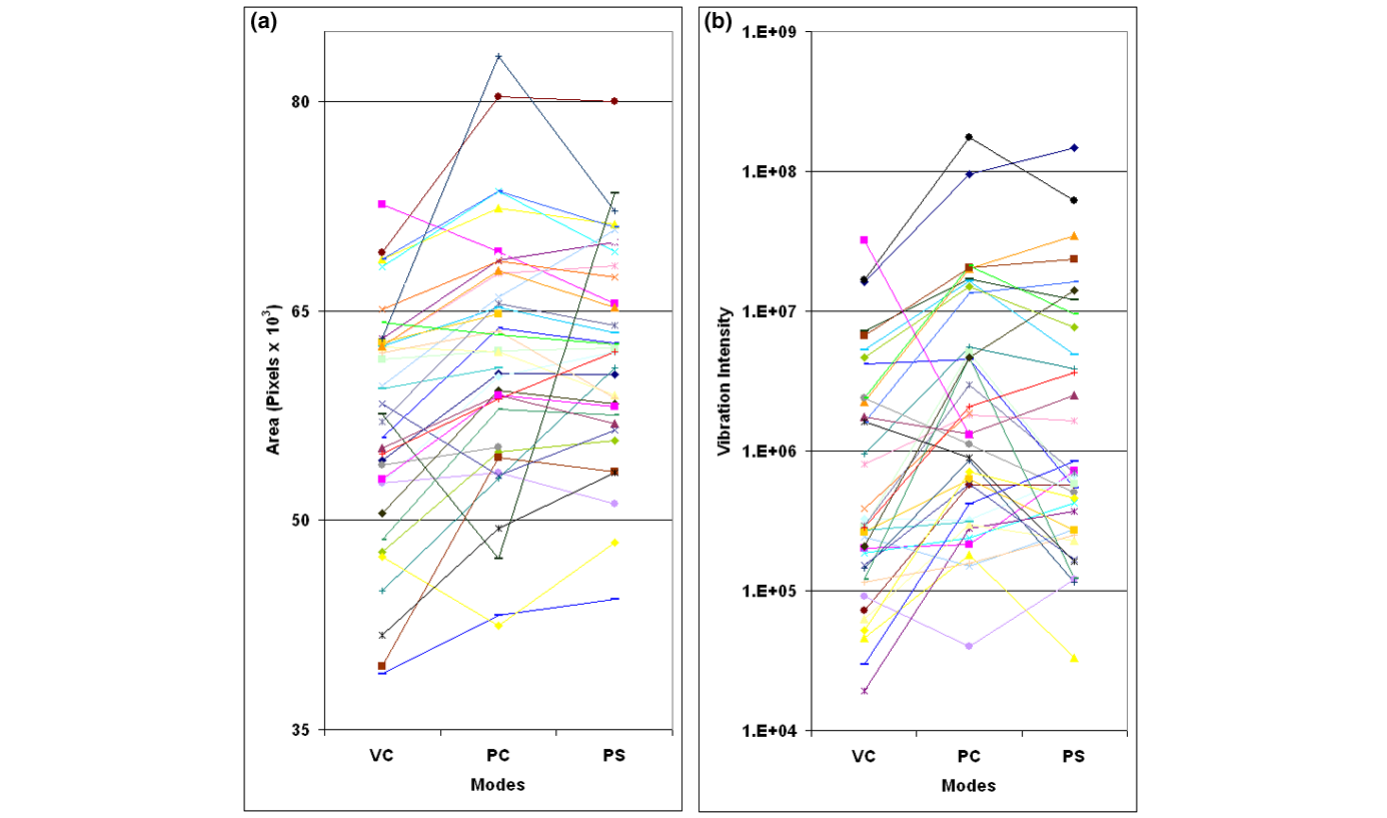
Mean area and vibration among individual patients. Mean areas of each patient **(a) **and mean vibration intensity values of each patient **(b) **on assist volume control (VC), assist pressure control (PC), and pressure support (PS) are presented.

Images and numeric vibration intensity values during maximal inspiration were analyzed (Figure 5a,b). Data from 4 to 10 MEFs obtained during one recording were averaged. The coefficient of variation (CV) was calculated for each set of MEFs, revealing rather low intra-patient variability (CV of less than 10% for 95% of the data and CV of less than 5% for 80% of the data). Furthermore, the lack of randomization did not create a notable effect as assessed by analysis of the subgroups of patients recorded in a sequence other than VC-PC-PS (*n *= 9) (data not shown).

**Figure 6:**
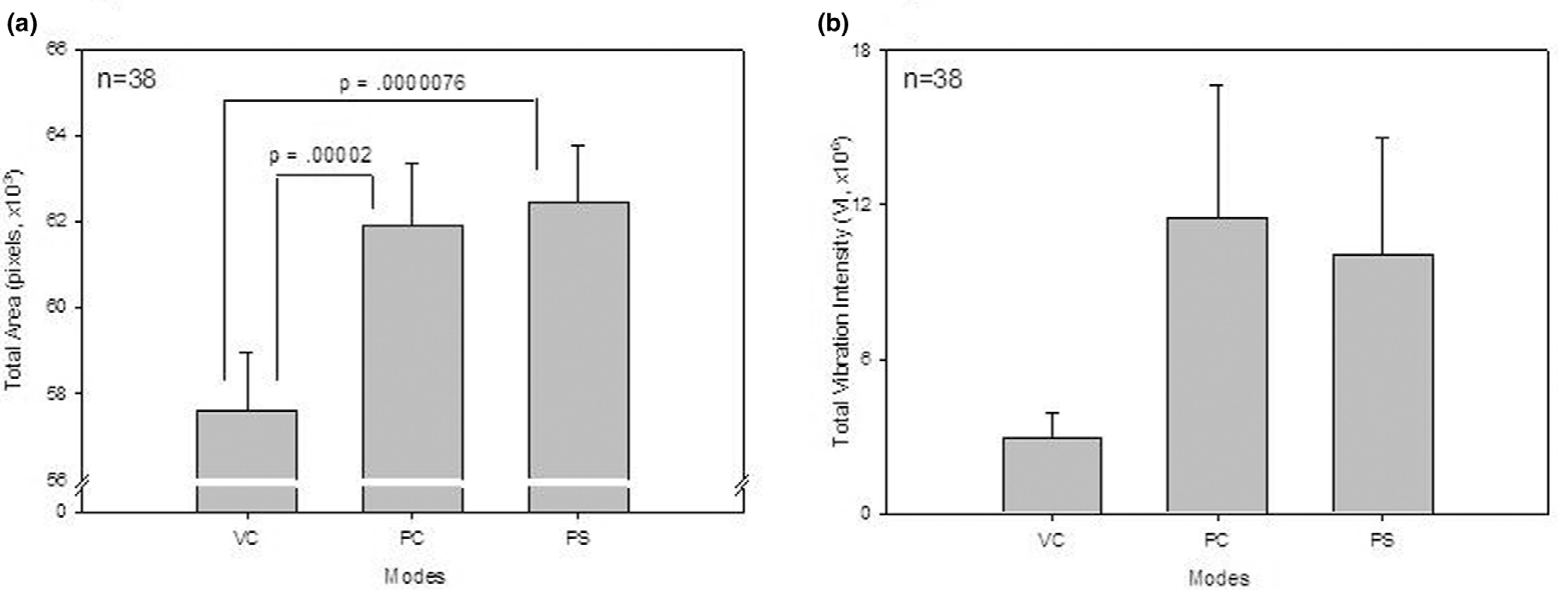
Total area and vibration intensity among modes. Mean total areas **(a) **and mean total vibration intensity values **(b) **on assist volume control (VC), assist pressure control (PC), and pressure support (PS) are presented. Total area differed significantly between VC and PC as well as between VC and PS. Data are presented as mean ± standard error of the mean.

**Figure 7:**
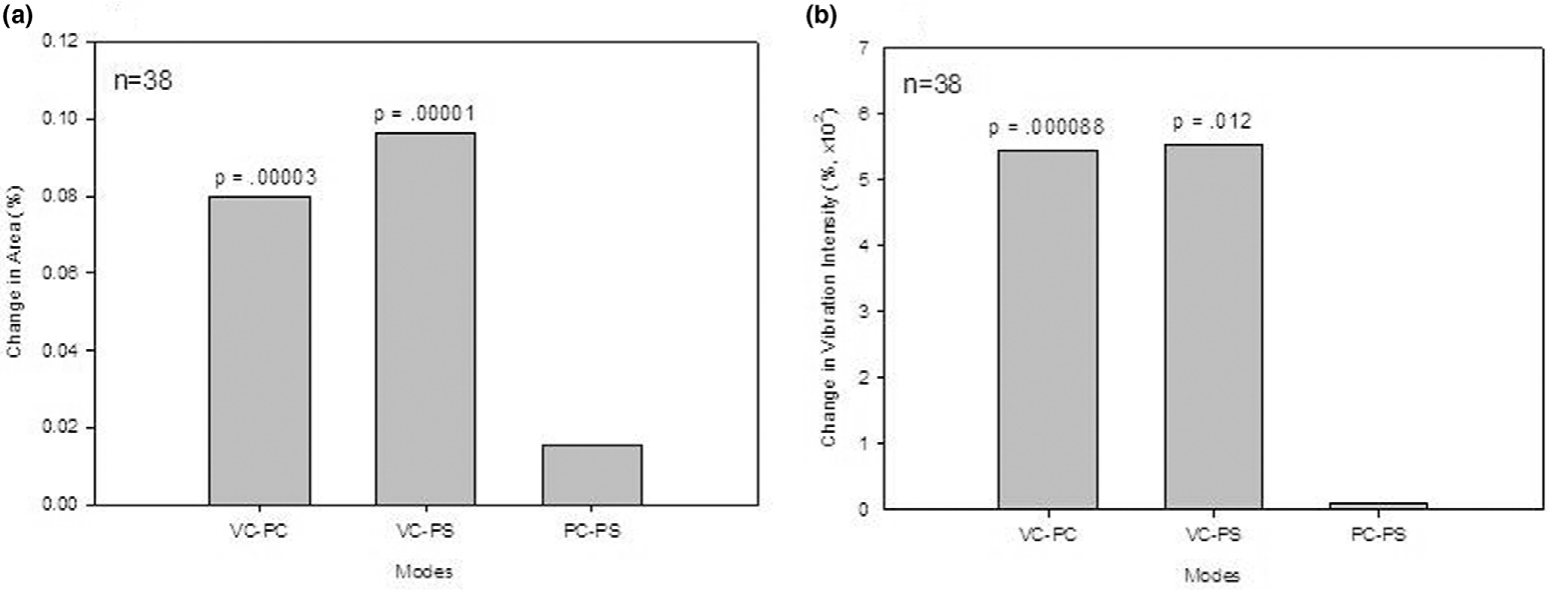
Distribution of area and vibration intensity between modes. Percentage changes in total areas **(a) **and percentage changes in total vibration intensity **(b) **between assist volume control (VC), assist pressure control (PC), and pressure support (PS) are shown. Percentage change in total area differed significantly between VC and PC modes as well as between VC and PS. VC to PC and VC to PS showed a significant difference in percentage change in total vibration intensity between modes.

The mean geographical area of the images recorded on PC and PS, compared to VC, revealed a significant overall increase in size (Figure 6a) (*p *< 0.001 for both). Each patient was used as his or her own control for comparing percentage change in area and total vibration signals. There was a significant percentage increase in geographical area (Figure 7a) and vibration (Figure 7b) from VC to PC and VC to PS (*p *< 0.02 for all). Although total vibration intensity was higher in PC and PS compared to VC, the difference was not significant (Figure 6b).

**Figure 8:**
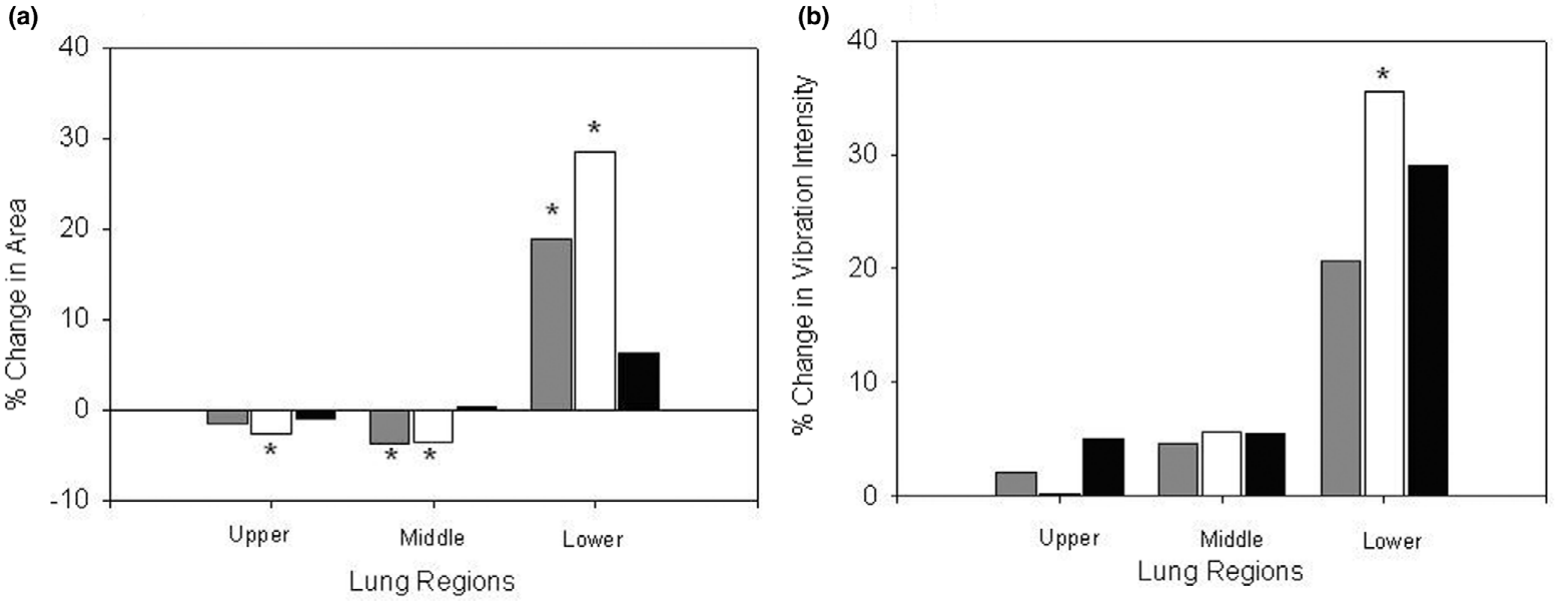
Redistribution of area and vibration intensity. Relative percentage changes in area **(a) **and relative percentage changes in vibration intensity **(b) **in different lung regions between assist volume control (VC), assist pressure control (PC), and pressure support (PS) are presented as mean percentage changes ± standard error. Gray represents VC-PC, white represents VC-PS, and black represents PC-PS. The asterisks indicate *p *values of less than 0.05, considered to be statistically significant. The relative percentage change in area in the middle and lower regions changed significantly from VC to PC and PS modes **(a)**. A difference in relative percentage change in vibration between VC and PS was observed in the lower lung region.

Regional area analysis demonstrated that the increase in the total area was due to the expansion of the lower lung region whereas areas in the upper and the middle regions decreased (Table 3). Assessment of relative percentage changes in areas revealed an increase in area in the lower lung regions and a decrease in the upper and middle regions (Figure 8a). When comparing VC to PC and to PS, the data showed a shift in image area away from the upper lung regions toward the lower.

**Table 3:** Regional area distribution

	Upper	Middle	Lower
	Mean ± SD	Mean ± SD	Mean ± SD
	
VC	40.4% ± 6.9%	36.0% ± 3.5%	23.6% ± 9.5%
PC	39.7% ± 6.3%	34.6% ± 3.3%	25.7% ± 8.9%
PS	39.5% ± 5.8%	34.6% ± 3.2%	25.8% ± 8.4%
*t *test	Upper ↓	Mid ↓	Lower ↑
VC-PC	0.170	0.00001^a^	0.002^a^
VC-PS	0.042^a^	0.0008^a^	0.004^a^
PC-PS	0.367	0.8598	0.536

^a^*p *< 0.05 indicates a significant difference in area distribution among VC and PS modes in all three regions. VC to PC mean regional area differs in the mid and lower lung regions. Tables 3 and 4 and their corresponding descriptions should be interpreted side by side. PC, assist pressure control; PS, pressure support; SD, standard deviation; VC, assist volume control; ↑ = increase; ↓ = decrease.

The regional vibration intensity values calculated from signals recorded in the three modes showed similar trends. There was a significant percentage increase in vibration intensity values in the lower regions (Table 4). The relative increase in vibrations in the lower region in PS versus VC was statistically significant (Figure 8b) (*p *< 0.05). Here again, a shift of vibration toward the lower lung regions was noted.

**Table 4:** Regional vibration intensity distribution

	Upper	Middle	Lower
	Mean ± SD	Mean ± SD	Mean ± SD
	
VC	48.7% ± 18.7%	40.5% ± 13.7%	10.8% ± 11.2%
PC	47.8% ± 19.4%	39.4% ± 13.7%	12.8% ± 15.4%
PS	48.5% ± 17.4%	39.5% ± 11.3%	12.1% ± 11.6%
*t *test	Upper	Mid	Lower ↑
VC-PC	0.709	0.610	0.107
VC-PS	0.411	0.747	0.027^a^
PC-PS	0.851	0.951	0.825

^a^*p *< 0.05 indicates a significant difference in vibration intensity distribution between VC and PS modes in the lower region. Tables 3 and 4 and their corresponding descriptions should be interpreted side by side. PC, assist pressure control; PS, pressure support; SD, standard deviation; VC, assist volume control; ↑ = increase.

**Figure 9:**
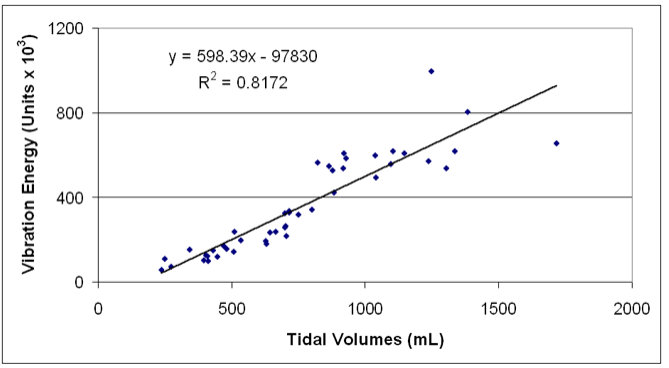
The effect of tidal volume/airflow on vibration intensity. There is a strong correlation and linear relationship between tidal volume and lung vibration intensity.

We demonstrated a strong correlation between the V_T _values and vibration energy in four healthy volunteers; the *R*^2 ^values were 0.81, 0.74, 0.78, and 0.82. Figure 9 displays the relationship between vibration and V_T_/airflow in one subject. Holding RR constant as V_T _increases, the total lung vibration measured with VRI increases linearly.

The mean peak airway pressures (± SD) in VC, PC, and PS were 28 ± 10, 25 ± 7, and 22 ± 8 mm H_2_O, respectively. These differences between the three modes were statistically significant by paired *t *test analysis (VC-PC < 0.02, VC-PS < 0.001, and PC-PS < 0.02).

## Discussion

The main finding of this study is that compared to VC, PS and (to a lesser extent) PC modes are characterized by an overall increase of geographical distribution of vibration in the lung. Furthermore, in PS and PC, vibration energy is shifted toward the lower lung regions when V_T _values are held constant. Two different computing methods were used to assess the regional distribution of vibration in the lungs: image analysis and raw numerical data calculation. In contrast to image analysis, the numerical method was not affected by normalization. The correlation of vibration energy and airflow in healthy lungs supports the premise that the increase in vibration in the lower lung regions in the subjects recorded within a two to five minute period is correlated strongly with an increase in flow in these regions. Because V_T _values were held constant, these results suggest that the distribution of airflow in the lower lung regions is greater in PC and PS compared to VC.

Two variables could contribute to a redistribution of airflow toward the lower lung regions in PS and PC versus VC: differences in inspiratory flow pattern and synchronization of patient diaphragmatic effort with the ventilator. PC and PS have a decelerating flow pattern with higher flow rates at the beginning of inspiration. This deceleration in flow is what may be characterized as 'pure' because it is driven by a pressure differential between patient and ventilator whereas the decelerating inspiratory waveform of VC (not used in this study) is determined by direct ventilator flow settings. The initial higher flow in PC and PS may serve to prime (quickly fill) the proximal airway, allowing more time for slower, more laminar flow to produce a more homogenous distribution of air to distal (lower) lung regions. Albeit controversially, some investigators have demonstrated that the decelerating flow waveform improves oxygenation compared to square waveform, even in the same mode (that is, square/rectangular versus decelerating VC) [5,9,16]. Our study offers a possible reason for such an improvement. The increase in total vibration observed in PS and (to a lesser extent) PC, compared to VC, may be due to the effect of higher initial flow on maximal vibration energy.

Patients who are mechanically ventilated may demonstrate ventilator dysynchrony, in which the desired breathing patterns do not match the ventilator and patient discomfort occurs [17,18]. Among the three modes, PS is the closest to spontaneous breathing in that the patient controls the length of inspiration and RR and, in turn, the V_T _and inspiratory flow rate are more adaptable to the patient's own ventilatory demand [19].

The physiologic explanation for the increase in vibration in the lower lungs during PS in our study could be the increase in diaphragm-generated negative intrapleural pressure during inspiration. Evidence has accumulated that diaphragmatic displacements during spontaneous and mechanical breaths are different. The increased use of muscles of inspiration in modes that are more amenable to this interaction, such as PC and PS (in which inspiratory flow is affected by the degree of inspiratory muscle activity), could produce increased vibrations in lower lung fields due to increased diaphragm activity. Because this effect was also observed in PC, in which the breaths above a set rate were not different than with VC, it is unlikely to be a 'triggering'-produced effect only. Although ventilator-triggered breaths were not differentiated from the patient-triggered breaths in VC and PC, the lack of difference in breaths above a set rate between these two modes supports this premise. In VC, ventilated patients have limited capability to produce effects on inspiration other than changing frequency (no changes in inspiratory flow with changes in inspiratory muscle activity). In PS and PC, increased diaphragm activity increases flow. Among the types of mechanical ventilation breaths tested here, PS most mirrors spontaneous breathing [20]. Spontaneous breaths are associated with a predominant movement of the posterior diaphragm, which contains more muscle fibers, whereas controlled mechanical breaths cause diaphragm displacement mainly in the anterior diaphragm [21-23]. Most of the lung is seated on this dorsal region of the diaphragm. It would be anticipated that diaphragm activity would be greatest with PS (greatest patient interaction), least with VC (least patient interaction with synchronous ventilation), and intermediate with PC (in which patient diaphragm activity can influence flow). This gradation of anticipated diaphragm activity is consistent with our results.

The square/rectangular waveform in VC, which maintains a fixed flow throughout the inspiration with higher flows at end-inspiration compared to PC and PS, leads to a higher peak airway pressure. Previous studies have also shown that a decelerating waveform results in lower peak airway pressures and higher mean airway pressures [9]. Peak airway pressure is achieved at end-inspiration in VC and is constant in PC. The lower peak airway pressure in PC reflects a lower inspiratory flow rate at end-inspiration when elastance is highest (largest lung volume). The initial loading of non-gas-conducting airways with the decelerating flow waveform followed by slower flow rates later in inspiration may lead to better distribution of airflow to the lower lung regions.

Our results are supported by recent studies that demonstrate that superimposed spontaneous breathing during airway pressure release ventilation redistributes tidal ventilation toward dependent lung regions just near the diaphragm [24]. This conclusion was derived using single photon emission tomography in the pig model. In another pig model experiment, it was demonstrated that spontaneous breathing reopens non-aerated lung tissue in dorsal juxtadiaphragmatic regions [25]. Our data reveal similar results in ICU patients by means of a different novel technique of imaging, featuring distribution of vibration as a surrogate of flow. VRI offers information at the bedside not previously available through other technologies and provides the potential to study the intensity and distribution of vibration within the lungs in real time. It is possible that VRI obtained in an individual patient could provide information on whether a particular distribution of vibration signified better overall ventilation or oxygenation in that patient.

### Study limitations

Physiologic effects other than distribution of vibration were not ascertained nor were outcome parameters obtained. The recordings were carried out in rapid succession in order to minimize variables such as changes in patient condition and sensor placement. The inter-patient variations in vibration intensities pose potential difficulties in analyzing data from different patients. To overcome this limitation for analysis of geographic area differences and total vibration energy among the three modes, each patient served as his/her own control and relative percentage change was analyzed over the group. The reason for this large difference is not yet characterized but was not correlated with body mass index in our study patients (*r *= 0.002, data not shown).

In this study, because not all ventilators used were capable of all waveform selections, we did not compare VC with decelerating inspiratory flow pattern to other modes. It would be useful, however, to compare PC to VC with decelerating waveform. Also, the fact that each recording is normalized to itself may result in an area of the images of similar vibration energy values to be gray for one patient and white for another. Although using each patient as his/her own control aids in alleviating this concern, this approach does not completely eliminate the potential confounding effect of normalization. The analysis of vibration intensity shifts, however, is not influenced by normalization and supports the findings of geographical surface area, making normalization an unlikely confounding factor. The MEF, which displays the peak inspiratory vibration, was selected as the frame providing the most information on the distribution of lung vibrations and on the overall lung condition. However, whether this is the most important period to analyze distribution of vibration remains to be determined. Moreover, the use of a heterogeneous group of ventilated patients with varied diagnoses may hide much greater effects in a subset of similar patients or different effects among patient subgroups, so our conclusions are of a general nature only.

Peak flow data were not collected in this study, which is a potential confounder given that data were not constant across the three modes. If inspiratory time is the same, to deliver the same V_T _would require higher peak flows at the start of inspiration in the pressure-targeted modes compared to VC. This may partly explain the higher vibration intensity observed in PC and PS. However, the fact that this change in flow pattern led to greater vibration intensity in lower lung regions still has clinical relevance. Multiple different ventilation types were used in this study. This may have had some influence on flow pattern and intensity. Ventilator specifications available make integration of this type of information into analyses difficult. However, common to all ventilators would be basic tenets of differences and similarities in flow 'patterns' among these three modes.

For technical reasons (need for vacuum sensors that must be free of contact with other objects), the recordings were carried out in the sitting position and would be expected to be different in the supine or intermediate (30° to 45°) position due to shift in fluid and gravity effect in blood flow. However, changes in vibration energy distribution between different modes of MV in the sitting position still have physiologic relevance and are of interest and potential clinical importance.

## Conclusion

In our study, pressure-targeted ventilation (PS more so than PC) shows a shift of vibration toward the lower, dependent lung regions compared to VC when V_T _is held constant. Synchronization with the ventilator, greater downward movement of the diaphragm, and decelerating flow waveform may be the physiologic explanation for the redistribution of vibration energy to lower lung regions in PS mode of MV. Further studies in the supine position are needed to correlate vibration intensity and distribution with oxygenation, ventilation, and clinical outcome.

## Key messages

• With V_T _held constant, PC and PS modes of MV, compared to VC, produce an increase in maximal inspiratory vibration energy in the lower lung regions.

• Better patient synchronization with the ventilator, greater downward movement of the diaphragm, and decelerating flow waveform are potential physiologic explanations for the redistribution of vibration energy to lower lung regions in pressure-targeted modes of MV.

• VRI is a novel non-invasive bedside technology that displays both a real-time structural and functional video of airflow-induced vibrations as well as total and regional graphs of vibration energy.

## Abbreviations

CV = coefficient of variation; FiO_2 _= fraction of inspired oxygen; ICU = intensive care unit; MEF = maximal energy frame; MV = mechanical ventilation; PC = assist pressure control; PEEP = positive end-expiratory pressure; PS = pressure support; RR = respiratory rate; SD = standard deviation; VC = assist volume control; VRI = vibration response imaging; V_T _= tidal volume.

## Competing interests

JEP and RPD have consultant agreements that include honoraria and stock options (no current monetary value) with Deep Breeze Ltd. Research personnel and materials for the VRI research program at Cooper University Hospital (Camden, NJ, USA) are funded partially by Deep Breeze Ltd. YAG is an employee of Deep Breeze Ltd. SJ, IC, CT, and SR declare that they have no competing interests.

## Authors' contributions

SJ, IC, CT, and SR carried out the VRI recordings. SJ, IC, CT, SR, and YG worked on the calculations of recordings. RPD, SJ, and IC drafted the manuscript. RPD and JEP participated in the design and coordination of the study and helped to draft the manuscript. All authors edited and approved the final manuscript.

## Supplementary Material

Additional file 1 An example of a VRI video recording of a healthy, 30-year-old, male non-smoker. L, left lung; R, right lung. Click here for file

Additional file 2 A VRI video recording of a mechanically ventilated female on assist volume control mode. Chest radiography reported pleural fluid in both lungs. L, left lung; R, right lung. Click here for file

Additional file 3 A VRI video recording of a mechanically ventilated female on assist pressure control mode. Chest radiography reported pleural fluid in both lungs. L, left lung; R, right lung. Click here for file

Additional file 4 A VRI video recording of a mechanically ventilated female on pressure support mode. Chest radiography reported pleural fluid in both lungs. L, left lung; R, right lung. Click here for file
